# Pan-class I  PI3-kinase inhibitor BKM120 induces MEK1/2-dependent mitotic catastrophe in non-Hodgkin lymphoma leading to apoptosis or polyploidy determined by Bax/Bak and p53

**DOI:** 10.1038/s41419-018-0413-4

**Published:** 2018-03-07

**Authors:** Anja Müller, Bernd Gillissen, Antje Richter, Anja Richter, Cindrilla Chumduri, Peter T. Daniel, Christian W. Scholz

**Affiliations:** 1 Department of Hematology, Oncology and Tumor Immunology, Charité - University Medicine Berlin, Campus Berlin-Buch, Lindenberger Weg 80, Berlin, 13125 Germany; 2 Department of Molecular Biology, Max Planck Institute for Infection Biology, Charitèplatz 1, Berlin, 10117 Germany; 3 Clinical and Molecular Oncology, Max Delbrück Center for Molecular Medicine, Berlin-Buch, 13125 Germany

## Abstract

Constitutive signaling of PI3K/Akt/mTOR plays a prominent role in malignant transformation and progression of B-cell non-Hodgkin lymphomas (B-NHL) underscoring the need for PI3K targeted therapies. The pan-class I PI3-kinase inhibitor BKM120 has shown preclinical activity in distinct malignancies and is currently tested in clinical trials. Intratumor heterogeneity is an intrinsic property of cancers that contributes to drug resistance and tumor recurrence. Here, we demonstrate that inhibition of PI3-kinases by BKM120 attenuates growth and survival of B-NHL cell lines by inducing mitotic arrest with subsequent induction of intrinsic apoptosis. BKM120-mediated downregulation of Cyclin A and activation of the CDK1/Cyclin B1 complex facilitates mitotic entry. In addition, concomitant BKM120-mediated upregulation of Cyclin B1 expression attenuates completion of mitosis, which results in mitotic catastrophe and apoptotic cell death. In Bax and Bak deficient B-NHL, which are resistant to BKM120-induced apoptosis, BKM120-induced mitotic catastrophe results in polyploidy. Upon re-expression of wt p53 in these p53 mutated cells, BKM120-induced polyploidy is strongly reduced demonstrating that the genetic status of the cells determines the outcome of a BKM120-mediated pathway inhibition. Mitotic catastrophe and unfavorable induction of polyploidy can be prevented in this setting by additional inhibition of MEK1/2 signaling. Combining MEK1/2 inhibitors with BKM120 enhances the anti-tumor effects of BKM120, prevents prognostic unfavorable polyploidy and might be a potential strategy for the treatment of B-NHL.

## Introduction

In B-cell non-Hodgkin lymphoma (B-NHL), gene amplification of the PI3K (phosphatidylinositol-4,5-bisphosphate 3-kinase) subunit p110α, or loss  of its antagonist PTEN (phosphatase and tensin homolog) facilitate constitutive activation of PI3K and its downstream targets Akt/PKB and mammalian target of rapamycin (mTOR), which is associated with malignant transformation, tumor progression, and resistance against chemo- and radiotherapy^1^. Transient activation of the PI3K/Akt/mTOR pathway mediates G1/S transition by controlling cell cycle regulators such as Cyclin D1. Constitutive Akt/PKB signaling, however, can bypass not only DNA damage-induced G1/S arrest but also G2/M checkpoint arrest^2,3^. Data from non-small cell lung carcinoma cell lines implicated that PI3K is essential for mitosis, as treatment with PI3K inhibitors  induces death by mitotic arrest, also termed mitotic catastrophe^4^.

Apoptosis can be abrogated by Akt/mTOR-mediated activation of anti-apoptotic members like Bcl-2 and Mcl-1 or inactivation of pro-apoptotic factors, such as caspase-9 and Bad^5–8^. Therefore, constitutive activation of the PI3K/Akt/mTOR pathway impedes tumor cell killing and constitutes therapy resistance. In addition, involvement of PI3K/Akt/mTOR signaling in the regulation of alternative cell death mechanisms, such as autophagy, mitotic catastrophe, and necroptosis has been shown^4,9^.

The pivotal role of PI3K/Akt/mTOR signaling in proliferation and survival of tumor cells nominates this pathway as a target for therapeutic intervention. Temsirolimus, a derivative of the mTORC1 inhibitor rapamycin, gained approval for the treatment of mantle cell lymphoma (MCL)^10^. However, the effects of temsirolimus monotherapy in B-NHL are limited^10,11^. Possible reasons are feedback signaling via mTORC2 or S6K/IRS-1, both restoring Akt/PKB activity^12–14^. This suggests that blockade of upstream PI3K signaling may circumvent feedback signaling and could be far more effective. NVP-BKM120 (BKM120), a novel pan-class I PI3K inhibitor, is currently tested in different clinical trials^15,16^. Here we demonstrate that BKM120 induces mitotic catastrophe in B-NHL cell lines, leading to apoptosis or polyploidy depending on the availability of functional Bax, Bak and p53. Mitotic catastrophe is triggered by BKM120-dependent activation of the CDK1/Cyclin B1 complex and concomitant upregulation of Cyclin B1 accompanied by a strong mitotic arrest. Concomitant inhibition of MEK1/2 signaling blocks Cyclin B upregulation, enhances favorable apoptosis in sensitive and blocks unfavorable polyploidy in resistant cell lines.

## Results

### BKM120 inhibits PI3K/Akt/mTOR signaling and has anti-proliferative activity in B-NHL cells

**Fig. 1:**
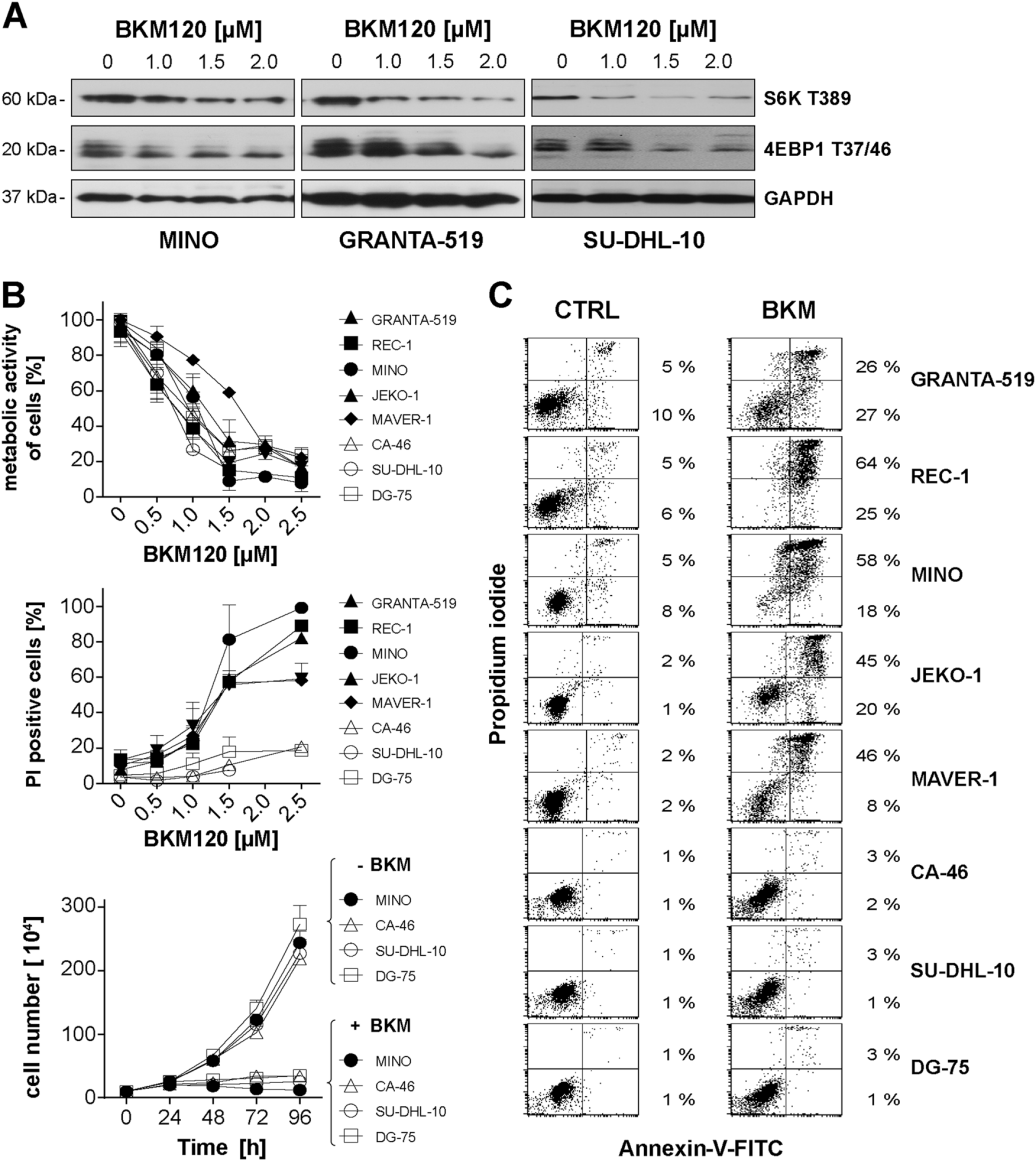
BKM120 affects Akt/mTOR signaling and has anti-proliferative as well as pro-apoptotic activity. **a** Phosphorylation of distinct PI3K downstream targets in MINO, GRANTA-519, and SU-DHL-10 after incubation with BKM120 at the indicated concentrations for 6 h. **b** Panel of non-Hodgkin lymphoma cell lines incubated with increasing concentrations of BKM120 for 72 h. Metabolic activity was assessed using the XTT assay (upper panel). Cell death induction was assessed using PI uptake (middle panel). Time dependent proliferation was assessed by counting the cell number of NHL cells treated with 2 µM and exclusion of dead cells by trypan blue staining (lower panel). All panels show mean and SD of three independent experiments. **c** Annexin-V-FITC/PI assay of the B-NHL cell line panel after incubation with 2 µM BKM120 for 72 h. Percentages refer to cells positive for both, PI and Annexin-V-FITC staining (upper right quadrant) and cells positive only for Annexin-V-FITC staining (lower right quadrant), respectively. Results are representative for three independent experiments

BKM120 abrogates PI3K signaling in three widely used B-NHL lines as indicated by decreases phosphorylation of downstream targets (Fig. 1a). BKM120 reduced S6K threonine 389 (T389) phosphorylation at concentrations of 1.5 µM in MINO and 1 µM in GRANTA-519 and SU-DHL-10 cells. Similarly, T37/46 phosphorylation of 4EBP1 was reduced in response to treatment with BKM120 at concentrations of 1.5 µM. Next, we examined the impact of BKM120 on the proliferation of B-NHL, including cell lines from mantle cell lymphoma (MINO, JEKO-1, REC-1, MAVER-1, and GRANTA-519), Burkitt lymphoma (CA-46, DG-75) and diffuse large B-cell lymphoma (SU-DHL-10). Treatment abrogated the metabolic activity of all cell lines (Fig. 1b, upper panel), but induced cell death only in MINO, JEKO-1, REC-1, MAVER-1, and GRANTA-519 cells (sensitive subgroup) as demonstrated by propidium iodide (PI) uptake (Fig. 1b, middle panel). In contrast, BKM120 did not induce cell death in CA-46, SU-DHL-10, and DG-75 cells (resistant subgroup). We also determined total cell numbers upon BKM120 treatment in the resistant cell lines compared to sensitive MINO control-cultures. Over time, cell numbers decreased in case of MINO and hardly increased in resistant cell lines (Fig. 1b, lower panel), demonstrating that BKM120 has an anti-proliferative effect on B-NHL cells.

### BKM120 induces intrinsic apoptosis in sensitive B-NHL cell lines

**Fig. 2:**
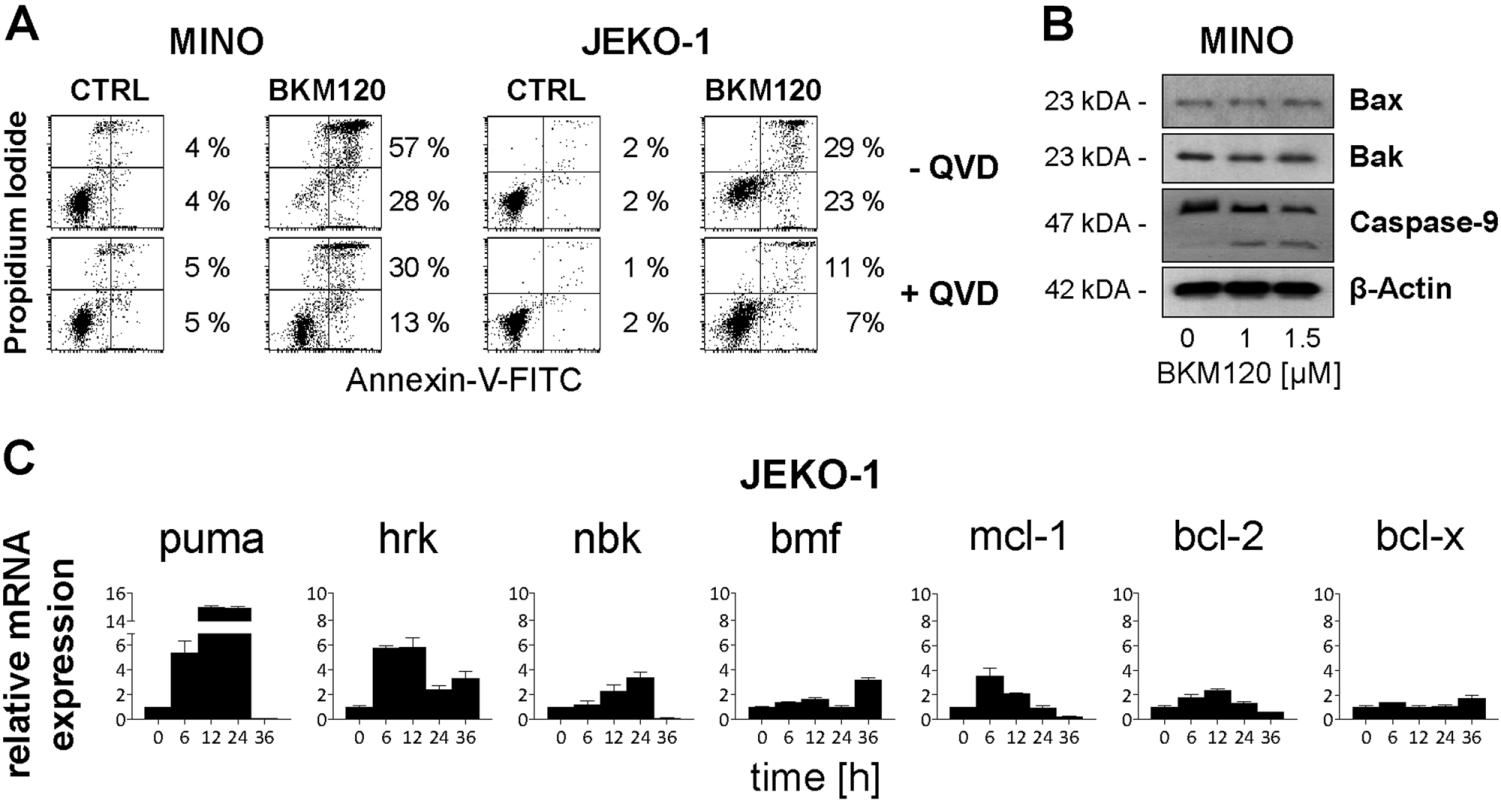
BKM120 kills cells via the intrinsic apoptosis pathway. **a** Annexin-V-FITC/PI assay in MINO and JEKO-1 cells in response to 2 µM BKM120 for 48 h and concomitant blockage of caspases with 10 µM QV-D-OPh. Percentages refer to cells positive for both, PI and Annexin-V-FITC staining (upper right quadrant) and cells positive only for Annexin-V-FITC staining (lower right quadrant), respectively. Results are representative for three independent experiments. **b** Measurement of the protein levels of Bax and Bak and cleavage/activation of caspase-9 in MINO cells in response to 1 µM and 1.5 µM of BKM120 incubated for 24 h. **c** Relative mRNA expression levels of Bcl-2 family members after incubation of JEKO-1 cells with 1.5 µM of BKM120 for the indicated time points [h]. Mean ± SD of three independent experiments

To test whether BKM120 induces apoptotic cell death in sensitive B-NHL cell lines, BKM120 treated cells were stained for phosphatidylserine exposure by Annexin-V-FITC binding and counterstained with PI. Flow cytometry analysis revealed, that incubation with 2 µM BKM120 for 72 h induced apoptosis in 53% of GRANTA-519, 89% of REC-1, 76% of MINO, 65% of JEKO-1 and 54% of MAVER-1 cells (Fig. 1c, bar graph in Supplement 1A). Consistent with a BKM120-resistant phenotype, CA-46, SU-DHL-10, and DG-75 cells were negative for Annexin-V-FITC and Annexin-V-FITC/PI (Fig. 1c), even at concentrations of up to 100 µM (Supplement 1B, 1C). Time escalation demonstrated most efficient induction of apoptosis after 48 h in MINO or 72 h in GRANTA-519 (Supplement 2A). Pretreatment with the pan-caspase inhibitor Q-VD-OPh reduced BKM120-induced phosphatidylserine externalization (Fig. 2a) and attenuated DNA fragmentation as demonstrated in MINO and JEKO-1 cells (Supplement 2B). BKM120 triggered a conformational change of pro-apoptotic Bax and Bak leading to the exposure of the respective N-terminus and activation of the proteins (Supplement 2C), which was accompanied by breakdown of the mitochondrial membrane potential (Supplement 2D) and processing of the initiator procaspase-9 (Fig. 2b), altogether indicating that BKM120 induces the Bax/Bak-dependent intrinsic apoptosis pathway.

We next analyzed whether BKM120 controls the transcription of BH3-only or anti-apoptotic Bcl-2 family members, which are known to regulate Bax/Bak activation. When analyzing mRNA expression levels in JEKO-1 cells, we observed a pronounced increase in mRNA expression of the BH3-only family members Puma and Hrk within 6 h post BKM120 treatment (Fig. 2c). In contrast, a weak and delayed upregulation of mRNA expression was detected for Bik/Nbk and Bmf. Among the anti-apoptotic Bcl-2 members, a transient increase of Mcl-1 mRNA expression was observed within 6 h, while Bcl-2 and Bcl-x_L_ expression were not affected (Fig. 2c).

### PI3K-inhibition by BKM120 induces mitotic catastrophe

**Fig. 3:**
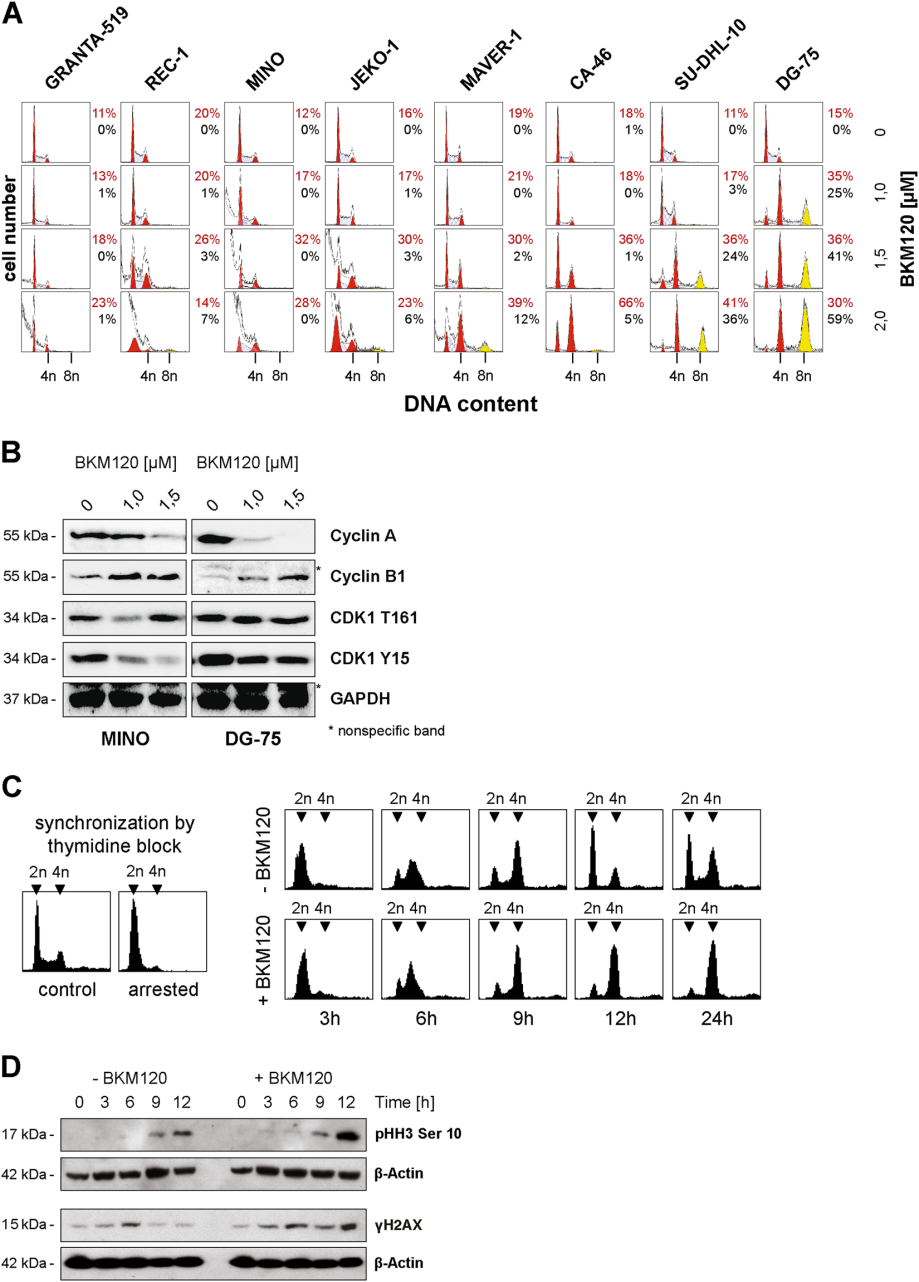
BKM120 induces mitotic arrest. **a** Cell cycle distribution of NHL cell lines incubated with the indicated concentrations of BKM120 for 72 h. Quantification with ModFit LT. Tetraploid cells are designated as 4n and hyperdiploid cells as 8n. Normal DNA content of 2n or 4n is colored in red, cells with hyperdiploid DNA content are yellow. Cells in S-phase are labeled with blue grid. Sub G1 cells are gray. Data are representative for three independent experiments. **b** Phosphorylation and expression status of cell cycle proteins in the two cell lines MINO and DG-75 after incubation with the indicated concentrations of BKM120 for 24 h. **c** DG-75 cells were synchronized by a double-thymidine block. Upon release from the thymidine block, DNA content of synchronized control cells and cells treated with 1.5 µM BKM120 was analyzed by flow cytometry. Representative histograms are shown. **d** Expression of the mitotic marker phospho-histone H3 (pHH3) Ser10 in synchronized DG-75 control and BKM120 treated cells was analyzed by Western blot analysis

Apoptosis is triggered in the context of attenuated proliferation and cell cycle arrest. Therefore, we examined the impact of BKM120 on cell cycle regulation. Treatment with 1.5 µM BKM120 for 72 h induced G2/M cell cycle arrest in all cell lines, irrespective of apoptotic sensitivity, i.e., in 18% GRANTA-519, 26% REC-1, 32% MINO, 30% JEKO-1, 30% MAVER-1, 36% CA-46, 36% SU-DHL-10, and 36% DG-75 cells (Fig. 3a). G2/M arrest was initiated as early as 6 h post BKM120 treatment and reached its maximum by 48 h and 24 h, as demonstrated in MINO and DG-75 cells, respectively (Supplement 3A).

Because the CDK1/Cyclin B complex regulates mitotic entry, phosphorylation of CDK1 was assessed in MINO and DG-75 cells. In both cell lines, CDK1 was phosphorylated at T161, which stabilizes binding of Cyclin B1 to CDK1. However, concurrent phosphorylation of Y15 (Fig. 3b), which prevents premature complex activation^17^, indicates inactivation of the complex. In the presence of BKM120, phosphorylation of CDK1 on T161 persisted, while phosphorylation of Y15 was reduced, thus demonstrating activation of CDK1/Cyclin B1 and implying mitotic entry. Furthermore, treatment with BKM120 caused downregulation of Cyclin A and upregulation of Cyclin B1 (Fig. 3b), indicating mitotic arrest because degradation of Cyclin A is a prerequisite for metaphase to anaphase transition and degradation of Cyclin B is required for mitotic exit^18^. Thus, BKM120 facilitates mitotic entry followed by mitotic arrest and mitotic catastrophe.

To address if BKM120 triggers premature mitotic entry, we synchronized DG-75 cells by a double-thymidine block and compared the DNA content of control and BKM120-treated cells upon release from the block (Fig. 3c). 6 h after release, control and BKM120-treated cells were in the S-phase (DNA content between 2n and 4n), 9 h after release in G2/M, showing synchronous passing of S-phase, G2-phase and mitotic entry. 12 h after release, control cells completed mitosis and re-entered the cell cycle, as indicated by the increase of cells with 2n. In contrast, BKM120 treated cells were arrested in mitosis, as demonstrated by cells with 4n. This finding was confirmed by immunoblot analysis of the mitotic marker phospho-histone H3 (pHH3) Ser10 (Fig. 3d). Expression of pHH3-Ser10 9 h after release was comparable between control and BKM120-treated cells, indicating no time differences in mitotic entry. A more pronounced pHH3 expression 12 h after release reflects the mitotic arrest of BKM120-treated cells. To examine whether BKM120 induces replication stress and whether DG-75 cells enter mitosis in the presence of DNA damage, expression of γH2AX was analyzed. Immunoblotting revealed no abundant γH2AX expression in BKM120-treated DG-75 cells entering mitosis. Twelve hours after release, γH2AX is slightly increased in BKM-treated cells, which might indicate mitotic stress and damage associated with mitosis failure. Overall we show that, despite downregulation of Cyclin A and upregulation of Cyclin B1, BKM120 does not induce replication stress or premature, uncoordinated mitotic entry.

### Apoptosis resistance and p53 deficiency trigger BKM120-dependent polyploidy

**Fig. 4:**
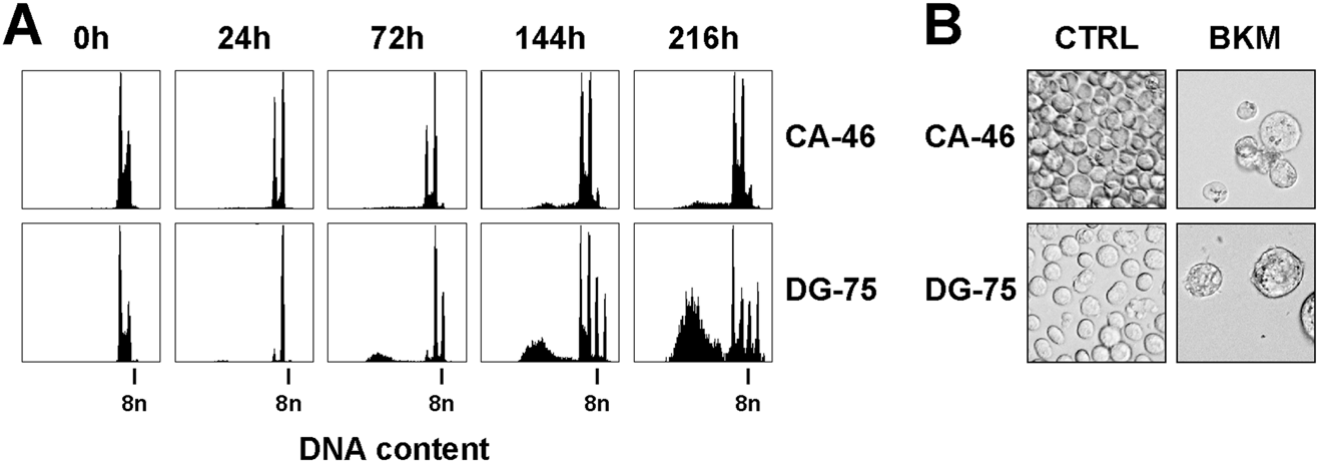
BKM120 induces polyploidy in apoptosis-resistant cells. Prolonged treatment of CA-46 and DG-75 with 1.5 µM of BKM120. Data are representative for three independent experiments. **a** DNA content and cell cycle distribution. **b** Light microscopy image  after 216 h

In apoptosis-resistant B-NHL cells, PI3K blockade by 2 µM BKM120-induced polyploidy (8n cells) in 5% of CA-46, 36% of SU-DHL-10 and 59% of DG-75 cells (Fig. 3a). Prolonged treatment with BKM120 for up to 216 h led to the formation of 16n cells in DG-75 (Fig. 4a). In addition, a massive increase of cell size was observed in CA-46 and DG-75 cells (Fig. 4b). This underscores that duplication of the DNA was triggered by multiple replications without mitotic cell division. To analyze if PI3K inhibitors other than BKM120 induce cell death or polyploidy in resistant B-NHL cells, we treated DG-75 cells with LY294002, BEZ235, and Wortmannin. In contrast to MINO cells which were used as controls, none of the drugs-induced cell death in DG-75 cells (Supplement 3B). Remarkably, only BKM120-induced polyploidy in DG-75 cells (Supplement 3C).

**Fig. 5:**
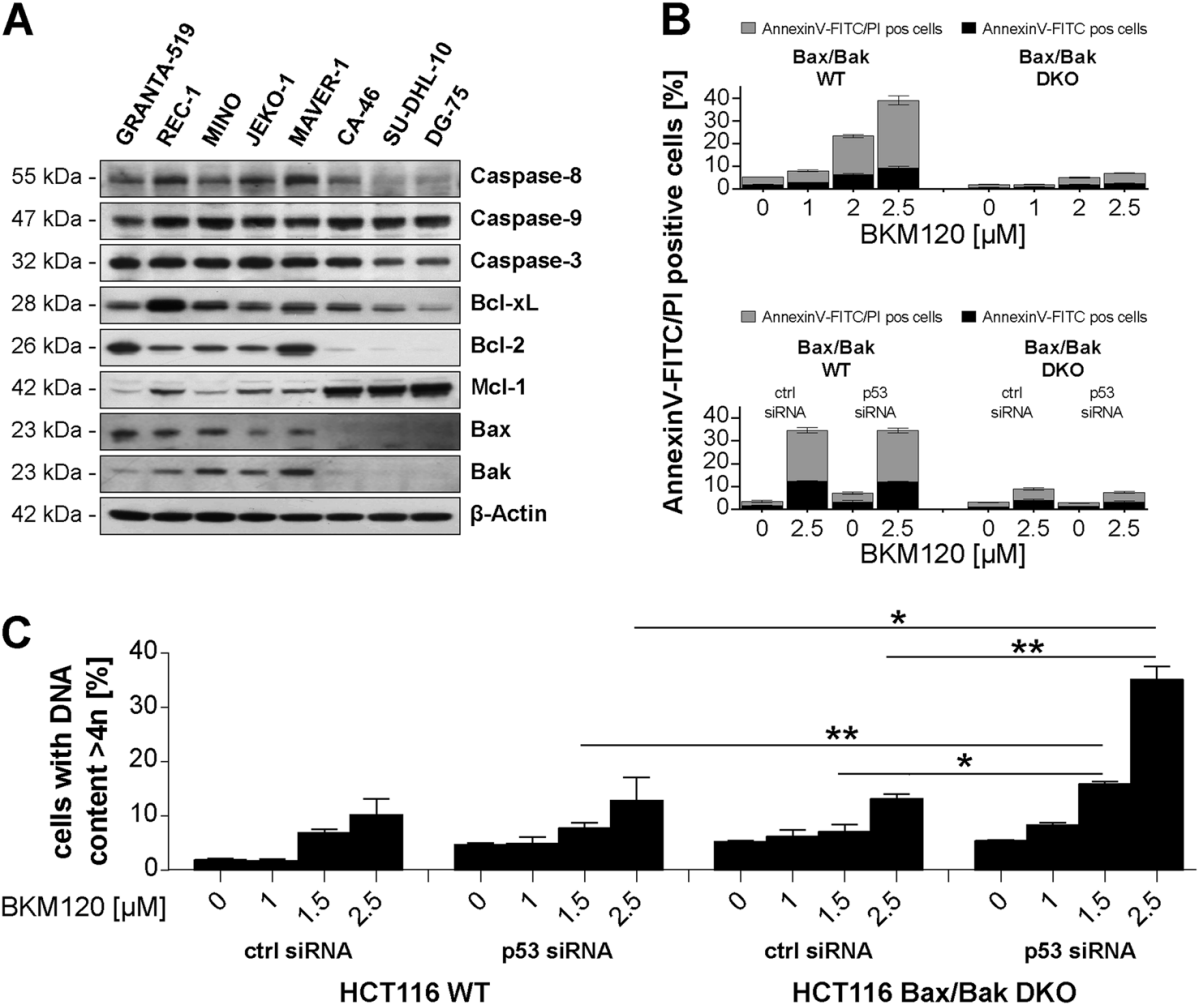
Concomitant Bax, Bax, and p53 deficiency determines polyploidy. **a** Basal protein expression levels of apoptotic regulators in a panel of NHL cell lines. **b** Annexin-V-FITC/PI assay of HCT116 WT or HCT116 Bax^−/−^/Bak^−/−^ cells incubated with increasing concentrations of BKM120 for 72 h (upper panel). Annexin-V-FITC/PI assay of HCT116 WT or HCT116 Bax^−/−^/Bak^−/−^ cells co-transfected with ctrl or p53 siRNA and incubated with 2.5 µM BKM120 for 72 h (lower panel). Data show mean ± SD of three independent experiments. **c** Polyploidy assay of HCT116 WT or HCT116 Bax^−/−^/Bak^−/−^ cells co-transfected with ctrl or p53 siRNA and incubated with increasing concentrations of BKM120 for 72 h. Data show mean ± SD of three independent experiments. *p < 0.05; **p < 0.005

Because our results suggested a link between BKM120-mediated polyploidy and apoptotic resistance, we next examined the expression levels of apoptotic regulators (Fig. 5a). Western blot analysis showed that procaspase-8 was variably expressed in all sensitive cell lines, barely detectable in the resistant cell lines SU-DHL-10 and DG-75, but clearly expressed in resistant CA-46 cells. Caspase-9 was expressed at comparable levels in all resistant and most sensitive cell lines, but slightly reduced in sensitive GRANTA-519 cells. Caspase-3 was slightly reduced in the resistant cell lines SU-DHL-10 and DG-75, but clearly expressed in the resistant cell line CA-46. Altogether there is no correlation between expression levels of caspases and sensitivity of cell lines.

Similarly no correlation was observed for the protein levels of the anti-apoptotic Bcl-2 family members Bcl-2 and Bcl-x_L_ and BKM120 resistance (Fig. 5a). To analyze if the high expression of Mcl-1 observed in all resistant cell lines is involved in resistance, we treated cells with Sabutoclax, a Bcl-2/Bcl-x_L_ inhibitor and potent Mcl-1 antagonist. Sabutoclax-induced apoptosis in MINO and MAVER-1 cells and sensitized MAVER-1 for BKM120-induced apoptosis (Supplement 4). In contrast, CA-46, SU-DHL-10, and DG-75 cells were resistant to Sabutoclax-induced apoptosis and inhibition of Mcl-1 failed to sensitize these cells for BKM120  induced cell death indicating that Mcl-1 is not linked to BKM120 resistance.

Interestingly, Bax and Bak were clearly expressed in all sensitive cell lines, whereas no Bax or Bak expression was observed in the three resistant cell lines (Fig. 5a). To analyze the role of Bax/Bak, we examined BKM120-induced apoptosis in the HCT116 cell line model system, where a Bax/Bak double knockout (Bax/Bak DKO) is available^19,20^. In HCT116 wild-type (WT) cells, BKM120-induced apoptosis in a dose-dependent manner (Fig. 5b, upper panel) with only a slight increase of polyploid cells at high-BKM120 concentrations (Fig. 5c). In HCT116 Bax/Bak DKO cells, BKM120-mediated apoptosis was attenuated (Fig. 5b, upper panel) but polyploidy was not increased compared to HCT116 WT cells. Thus, Bax/Bak deficiency incurs apoptotic resistance but is not sufficient to facilitate BKM120-mediated polyploidy.

Because all three B-NHL cell lines that developed BKM120-mediated polyploidy carry p53 mutations^21^ (http://p53.iarc.fr/CellLines.aspx), we next assessed the impact of additional siRNA-mediated p53 downregulation (Supplement 5A) on BKM120-induced polyploidy. Neither in HCT116 WT nor in Bax/Bak DKO cells downregulation of p53 did influence BKM120-mediated apoptosis (Fig. 5b, lower panel). However, in contrast to HCT116 WT cells, p53 downregulation lead to the induction of BKM-mediated polyploidy in HCT116 Bax/Bak DKO cells (Fig. 5c). This demonstrates that BKM120-dependent polyploidy requires the concomitant loss  of  Bax/Bak and p53. HCT116 cells harbors an activating KRAS mutation, which increases signaling through the MEK/ERK pathway indicated by constitutive MEK1/2 phosphorylation (Supplement 5B). Upon BKM120 treatment MEK1/2 phosphorylation is, like Cyclin B1 expression, not further increased, and  CDK1 Y15 phosphorylation is  reduced. However, upon BKM120 treatment HCT116 cells essentially reflect BKM120-treated B-NHL cells (activated MEK1/2, Cyclin B1 expression, reduced CDK1 Y15 phosphorylation).

**Fig. 6:**
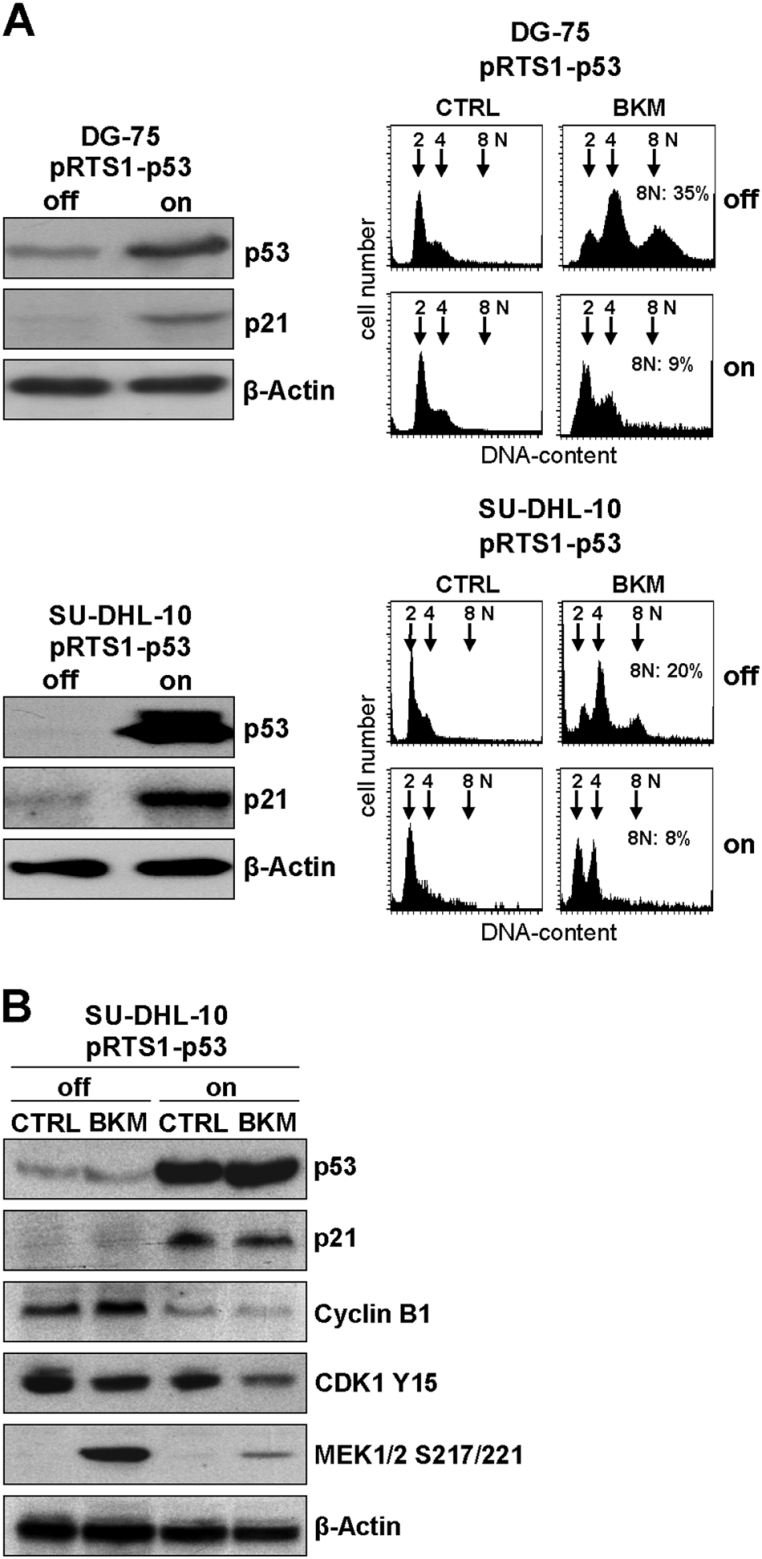
Re-expression of wt p53 inhibits induction of polyploidy by BKM120. DG-75 and SU-DHL-10 cells were transfected with pRTS1-p53, an episomal vector system for regulated expression of wt p53. **a** Western blot analysis confirmed overexpression of p53 under “on” condition, which is accompanied by increased p21 expression (left). FACS analyses of cellular DNA contend in resistant SU-DHL-10 and DG-75 revealed induction of polyploidy (cells with 8n, top right panels) by BMK120 treatment, which is strongly decreased by concomitant re-expression of p53 (right panels). **b** Western blot analysis of SU-DHL-10 cells revealed BKM120-induced increased Cyclin B1 expression, MEK1/2 activation and decreased CDK1 Y15 phosphorylation in the absence of p53. In p53 re-expressing SU-DHL-10 cells, Cyclin B1 expression is almost completely suppressed, independently of BKM120 treatment. Furthermore activation of MEK1/2 by BKM120 is strongly reduced upon p53 expression

To verify the results in B-NHL cell lines, p53 was re-expressed in resistant DG-75 and SU-DHL-10 cells by using pRTS1-p53, an episomal one-vector system^22^ allowing doxycycline-induced, conditional expression. Dox-induced expression (on condition) of p53 was confirmed by Western blot analysis (Fig. 6a left panels). Expression of p53 was accompanied by increased expression of the cyclin-dependent kinase inhibitor p21^CIP1/WAF1^, indicating functional p53 downstream signaling. As shown before, BKM120 caused polyploidy in resistant DG-75 and SU-DHL-10 cell lines (Fig. 6a right panels). Upon re-expression of p53, however, polyploidy was strongly reduced. In p53 re-expressing SU-DHL-10 cells CDK1 Y15 phosphorylation is still reduced upon BKM120 treatment. However, p53 re-expression almost completely suppressed expression of Cyclin B1 and inhibited BKM120-mediated activation of MEK1/2 (Fig. 6b), indicating that besides upregulation of p21, reduced MEK1/2 activation and suppression of Cyclin B1 contribute to the inhibition of BKM-induced polyploidy by p53.

### Polyploidy is MEK1/2 dependent

**Fig. 7:**
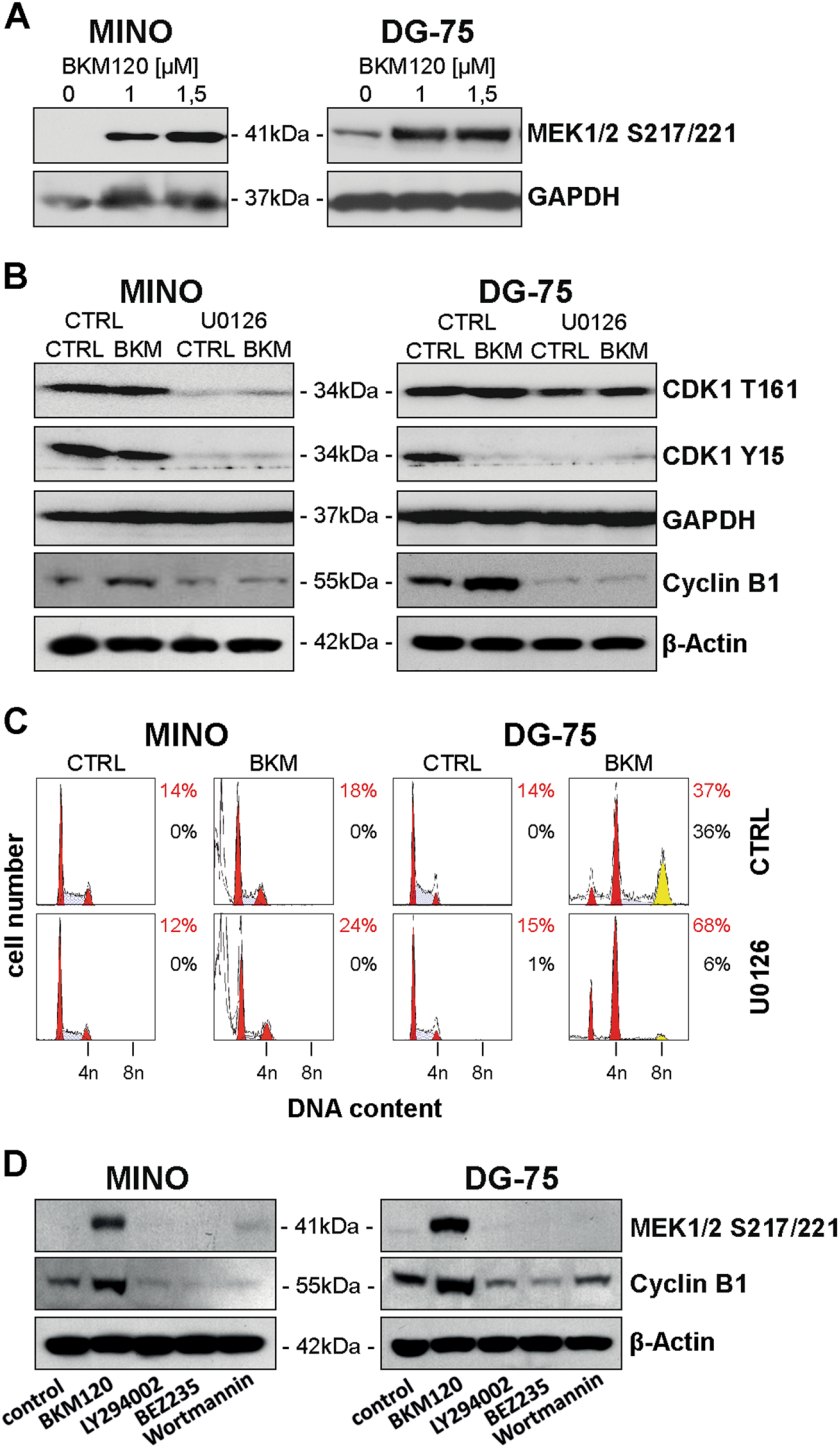
BKM120-induced mitotic arrest and polyploidy requires MEK1/2. **a** Expression of phosphorylated MEK1/2 in MINO and DG-75 after treatment with increasing concentrations of BKM120 for 3 h. **b** Expression of Cyclin B1 and phosphorylated CDK1 in MINO and DG-75 cells pretreated with 50 µM U0126 for 4 h and subsequently incubated with 1 µM BKM120 for 24 h. **c** Measurement of cell cycle distribution and polyploidy of MINO and DG-75 cells pretreated with 50 µM U0126 for 4 h and subsequently incubated with 1 µM BKM120 for 72 h. Quantification with ModFit LT. Data shown are representative for two independent experiments. **d** WB analysis of MEK1/2 phosphorylation and Cyclin B1 expression in MINO and DG-75 cells upon treatment with 1.5 µM BKM120, 20 µM LY294002, 5 µM BEZ235, or 100 µM Wortmannin

To analyze the impact of BKM120 on MAPK signaling, we explored the activation of MEK1/2 in MINO and DG-75 cells. BKM120 treatment  induced phosphorylation of MEK1/2 at S217/221 (Fig. 7a). To examine whether MAPK signaling is important for BKM120-induced CDK1/Cyclin B1 activation and mitotic catastrophe, MEK1/2 activity was abrogated by U0126. In both cell lines, pretreatment with U0126 attenuated phosphorylation of CDK1 at T161 and Y15 (Fig. 7b). Additional administration of BKM120 did not affect T161 or Y15 phosphorylation. Hence, U0126 abrogated BKM120-induced activation of CDK1/Cyclin B1 and mitotic catastrophe by regulating CDK1/Cyclin B1 assembling upstream of BKM120. Furthermore, while BKM120 induces Cyclin B1 expression, concomitant treatment with BKM120 and U0126 abolishes this effect, suggesting that MEK1/2 is necessary for BKM120-mediated Cyclin B1 upregulation (Fig. 7b).

To test whether concomitant inhibition of MEK1/2 affects BKM120-mediated apoptosis, MINO cells were treated with U0126 and BKM120. Administration of BKM120 alone induced apoptosis, while U0126 alone did not (Supplement 5C). Interestingly, U0126 significantly sensitized MINO cells for apoptosis-induced by BKM120. When assessing the effect of combined treatment on polyploidy in resistant DG-75 cells, treatment with U0126 alone did not affect ploidy of DG-75 cells, while co-culture with U0126 and BKM120 reduced the amount of polyploid cells (8n) from 36 to 6% (Fig. 7c). As  pretreatment with U0126 clearly reduced BKM120-induced polyploidy, MEK1/2 controls BKM120 triggered mitotic arrest and polyploidy.

To examine why other inhibitors of PI3K fail to induce polyploidy, we analyzed activation of MEK1/2 and Cyclin B1 expression upon treatment of MINO and DG75 with LY294002, BEZ235, and Wortmannin. In contrast to BKM120, none of the inhibitors  induced MEK1/2 activation or Cyclin B1 upregulation (Fig. 7d) and therefore most likely fail to cause polyploidy.

### BKM120-mediated events are reversible

**Fig. 8:**
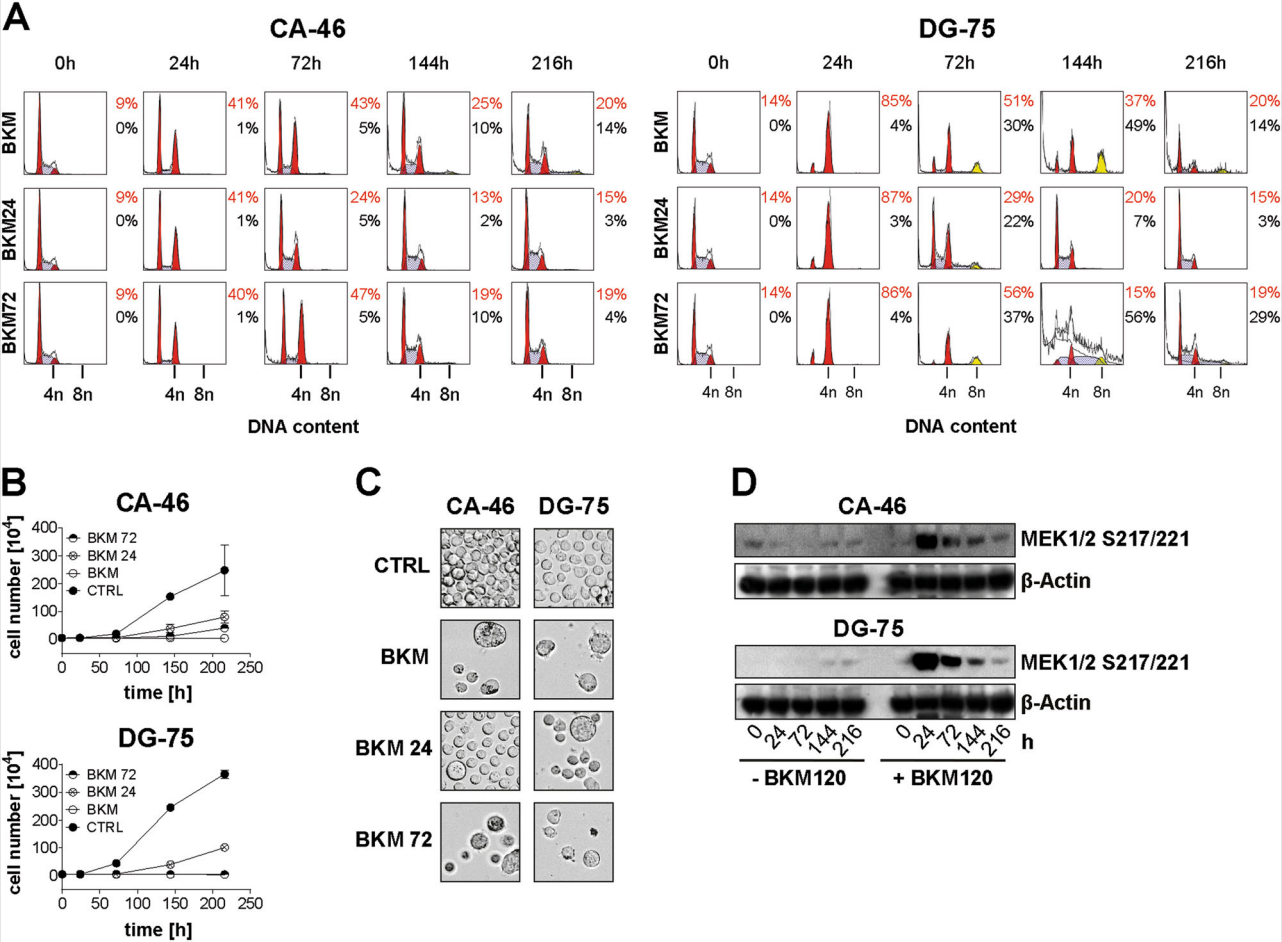
BKM120-mediated processes are, in part, reversible. CA-46 or DG-75 cells temporarily treated with 1.5 µM BKM120. Reculture in media after 24 h (BKM24) or 72 h (BKM72) or full length treatment for 216 h (BKM). **a** Cell cycle analysis was assessed by flow cytometry and analyzed with ModFit LT. Data shown are representative for three independent experiments. **b** Proliferation was measured by counting the cell number and counterstaining dead cells with trypan blue. Data shown are mean ± SD of three independent experiments. **c** Light microscopy image  of cells treated with BKM120 for 216 h. **d** WB analysis of phosphorylated MEK1/2 upon long-term treatment of CA-46 and DG-75 cells with 1.5 µM BKM120

To examine the sustainability of mitotic catastrophe and polyploidy, transient BKM120 treatment was assessed in CA-46 and DG-75 cells. Both cell lines were incubated with BKM120 for 24 h or 72 h and re-cultured in medium up to a total of 216 h (Fig. 8a). Control cells were incubated with BKM120 for 216 h. In CA-46 cells, the highest rate of BKM120-dependent mitotic cells (43%) was observed after 72 h of incubation (Fig. 8a, CA-46, upper panel). Removal of BKM120 after 24 h, attenuated the amount of mitotic cells and prevented polyploidy (Fig. 8a, CA-46, middle panel). When BKM120 was removed after 72 h, cells recovered from mitotic arrest and polyploidy after 216 h (Fig. 8a, CA-46, lower panel). In DG-75 cells, BKM120  induced mitotic arrest in 85% of the cells after 24 h and polyploidy in 49% of the cells after 144 h (Fig. 8a, DG-75 upper panel). Removal of BKM120 after 24 h, enabled DG-75 cells to recover from mitotic arrest and polyploidy after an overall time of 216 h (Fig. 8a, DG-75 middle panel). When BKM120 was removed after 72 h, mitotic cells decreased to 19% and polyploid cells decreased to 29% after continued incubation for 216 h (Fig. 8a, DG-75 lower panel). Removal of BKM120 after short-term treatment for 24 h enabled both cell lines to overcome growth inhibition and to restore proliferation (Fig. 8b). In contrast, when BKM120 was removed after 72 h of incubation, only CA-46 cells were capable to restart proliferation. Interestingly, continued treatment with BKM120 for 216 h caused the formation of giant cells, a hallmark of polyploidy, while strongly decreasing the amount of normally sized cells in both cell lines (Fig. 8c). Short-term incubation with BKM120 for 24 h, but not 72 h, increased the amount of normally sized cells. Giant cells, however, were still detectable. BKM120 strongly induced phosphorylation of MEK1/2 at S217/221, necessary for BKM120-induced polyploidy, in both cell lines upon 24 h of treatment (Fig. 8d). Despite the presence of BKM120, phosphorylation of MEK1/2 gradually decreased over time, which might explain reduction of mitotic arrest and polyploidy upon prolonged BKM120 treatment.

## Discussion

Pan-PI3K inhibitors impede proliferation and induce cell death in various B-NHL cell lines and are currently tested in clinical trials or already gained approval^23–26^. Here we show that BKM120, a recently identified PI3K inhibitor, effectively inhibited PI3K-pathway signaling, abrogated proliferation, and induced G2/mitotic arrest in B-NHL cell lines. The reported cell cycle effects of PI3K inhibition are predominantly G1 arrest, but block of G2/M transition has also been described^2,27,28^. BKM120 is known to induce G2/M arrest in many cell lines^29–32^ but can also arrest cells in the G1 phase^33,34^, indicating that downstream effects of PI3K inhibition depend on the molecular background of the cells. Furthermore, some non-specificity among the inhibitors might be responsible for the different outcome, e.g., LY294002 not only inhibits class I PI3Ks but also other PI3K-related kinases like ATM, ATR^35,36^ and DNA-PK^37^. In addition to G2/mitotic arrest and in contrast to other PI3K inhibitors, BKM120 induces polyploidy in apoptosis-resistant B-NHL cell lines. This effect clearly depends on MEK1/2 activation by BKM120. Activated MEK1/2 might induce G1/S transition with subsequent G2/mitotic arrest due to BKM120-mediated PI3K inhibition.

BKM120 treatment caused activation of the mitotic checkpoint regulator CDK1/Cyclin B1. Phosphorylation of CDK1 at T161 stabilizes binding to Cyclin B^17^, while concurrent phosphorylation at T14 and Y15 maintains CDK1/Cyclin B inactive until conditions for mitosis are set. In the B-NHL cell lines tested, BKM120 reduced inhibitory Y15 but not activating T161 phosphorylation, thereby empowering CDK1/Cyclin B and promoting mitosis. Moreover, we observed that BKM120  induced the downregulation of Cyclin A and upregulation of Cyclin B1. We show here for the first time that BKM120-mediated induction of Cyclin B1 depends on MEK1/2. However, the exact mechanism of MEK1/2 activation and Cyclin B1 upregulation by BKM120 needs to be established.

Cyclin A degradation is required for metaphase–anaphase transition and mitotic progression^38,39^, whereas degradation of Cyclin B is required for completion of mitosis^18^. This substantiates that BKM120 facilitates mitotic entry and concomitantly prevents completion of mitosis resulting in mitotic catastrophe. While a precise definition for mitotic catastrophe is still missing, current knowledge indicates that irregularities during mitosis, such as unrepaired DNA damage or impaired spindle assembly, lead to mitotic catastrophe and facilitate distinct forms of programmed cell death^40^. In B-NHL cells, BKM120-mediated mitotic catastrophe resulted in apoptosis via the Bax/Bak dependent intrinsic pathway. Our quantitative RT-PCR analysis suggests that transcriptional upregulation of the BH3-only proteins Puma and Hrk participate in BKM120-mediated apoptotic signaling. While Puma is a well-known target of the PI3K/Akt pathway, there has been little evidence for PI3K-dependent Hrk expression to date^41,42^. Despite strong expression of Mcl-1, Sabutoclax did not sensitize resistant cell lines for BKM120-induced apoptosis. This indicates that Mcl-1 expression is not linked to BKM120 resistance but might be related to functions of Mcl-1 not involved in the regulation of apoptosis, e.g., regulation of mitochondrial fusion, ATP production, regulation of ER Ca^2+^-transport systems^43–45^ and inhibition of autophagy^46,47^.

Polyploidization, as shown here for BKM120-treated resistant B-NHL cell lines, is a known feature of cells that have evaded the apoptotic death program of mitotic catastrophe^21,40,48,49^. The inability of CA-46, SU-DHL-10. and DG-75 cells to undergo apoptotic cell death results from the loss  of  Bax and Bak expression, which also triggers polyploidy in response to substances like paclitaxel or nocodazole^21,50,51^. In addition to Bax and Bak, polyploidy induced by BKM120 requires loss  of  p53, as demonstrated here in HCT116 Bax/Bak DKO cells after downregulation of p53. Furthermore, re-expression of p53 in B-NHL cells, which is accompanied by upregulation of p21, reduced MEK1/2 activation and suppression of Cyclin B1, inhibited BKM120-induced polyploidy. Interestingly, a combined inactivation of p53 and Bax has been shown to result in a very poor disease prognosis in B-CLL and solid tumors^52,53^. Furthermore, as DNA ploidy appears to have a negative impact on prognosis in general and on the tendency to metastasize in particular^54,55^, BKM120-mediated formation of polyploid cells may have therapeutic relevance as loss  of  Bax and Bak is observed in high-grade NHL and also contributes to NHL resistance against rituximab and chemotherapy^56,57^.

The inverse relationship between apoptosis and polyploidy reveals a decision point between mitosis progression, apoptosis and endoreplication^21,58,59^ and introduces polyploidy as an alternative pathway when apoptosis and cell cycle progression are obstructed^21,49^. This is, as shown here for B-NHL cells, accompanied by massive increase in cell size and the formation of giant cells, a prominent morphological characteristic of cells undergoing mitotic catastrophe. While underlying mechanisms are still unclear several principles such as endoreplication or endocycling are discussed, and endocycling has been specifically observed in p53 deficient tumor cells^50^.

Our data demonstrated BKM120-mediated activation of MEK1/2 and induction of Cyclin B1 expression. MEK1/2 inhibition abrogated BKM120-induced expression of Cyclin B1 which can affect BKM120-mediated polyploidy in two ways. Reduced Cyclin B levels might prevent mitotic entry, thereby arresting cells at the G2 checkpoint, which would inhibit mitotic catastrophe and subsequent induction of polyploidy. However, degradation of Cyclin B1 by anaphase-promoting complex/cyclosome is also required for mitotic exit and completion of the cell cycle. Reduced Cyclin B levels may therefore enable cells, which have escaped mitotic arrest or are already in mitosis at time of treatment, to successfully complete mitosis and reenter the cell cycle thereby inhibiting induction of polyploidy. In line with this, MEK1/2 activation was reported to correlate with ploidy and endomitosis in megakaryocytes^60^. In apoptosis sensitive cell lines, however, combination of BKM120 with U0126 synergistically enhanced apoptosis, which is known from other PI3K and MAPK inhibitors^61^. In contrast, BKM120-resistant cells undergo cell death after prolonged treatment by a caspase-independent mechanism (data not shown). Interestingly, BKM120 can induce autophagy besides apoptosis^16,62^. However, it has been shown that blocking of MEK/ERK signaling attenuates BKM120-induced autophagy^63^, making induction of autophagy after treatment of cells with BKM120 and U0126 unlikely. Whether cells alternatively die through a necroptotic form of cell death as shown before in other lymphoma therapy settings^64,65^, requires further analysis.

In conclusion, pan PI3K inhibition with BKM120 has anti-tumor potential for the treatment of B-NHL. Furthermore, combination with MEK1/2 inhibitors appears to enhance anti-tumor effects and to prevent prognostic unfavorable polyploidy in vitro. Currently running trials in advanced non-small Cell Lung Cancer combining the MEK inhibitor MEK162 with the PI3Kα inhibitor BYL719 (NCT02276027) or the MEK inhibitor Selumetinib with mTOR inhibitor AZD2014 (NCT02583542) underscore their therapeutic potential.

## Materials and methods

### Cell culture

The NHL cell lines JEKO-1, GRANTA-519, REC-1, MAVER-1, CA-46, DG-75, and SU-DHL-10 were purchased from DSMZ (Deutsche Sammlung von Mikroorganismen und Zellkulturen GmbH, Braunschweig, Germany), while MINO was obtained from ATCC (American Type Culture Collection, Manassas, VA, USA). The colon carcinoma cell line HCT116 wild type (HCT116 WT) was obtained from Dr. Bert Vogelstein (Johns Hopkins Cancer Center, Baltimore, MD, USA) and the HCT116 Bax/Bak double knockout (HCT116 Bax/Bak DKO) were kindly provided by Dr. Richard J. Youle (National Institutes of Health, Bethesda, MD, USA). All lymphoma cell lines were cultured in RPMI supplemented with 10% (GRANTA-519, REC-1, MINO) or 20% FCS (JEKO-1, MAVER-1) and 100 U/ml penicillin plus 100 µg/ml streptomycin. The HCT116 WT and HCT116 Bax/Bak DKO were cultured in DMEM supplemented with 10% FCS and 100 U/ml penicillin plus 100 µg/ml streptomycin.

### Inhibitor treatment

The pan-class I PI3K inhibitor BKM120 was obtained from Novartis (Novartis AG, Basel, Switzerland), solved in DMSO and stored in 10 mM stocks at −80 °C. For inhibitor treatment, cell lines were seeded at a denstiy of 0.5 × 10^5^–1 × 10^5^ cells in 24 wells (HCT116 cells at 1 × 10^5^ in 6 wells) and incubated at 37 °C overnight. Diluted inhibitor was added at the appropriate concentration and cells were incubated for the indicated time. MEK1/2 activity was blocked with the inhibitor U0126 from Calbiochem (Merck KGaA, Darmstadt, Germany). Pan-caspase inhibition was performed with QV-D-OPh from Calbiochem (Merck KGaA, Darmstadt, Germany). Blockade of MEK1/2 or caspases was induced by addition of the according inhibitor 4 h prior to BKM120 treatment.

### Metabolic activity assay

Proliferation was analyzed by the use of the cell proliferation kit II (XTT) from Roche (Basel, Switzerland) employing a Tecan Spectra reader (SLT, Crailshaim, Germany).

### Proliferation assay

Cells were treated with or without BKM120 and proliferation was analyzed by counting viable cells by use of a Neubauer chamber. Dead cells were identified by trypan blue staining and were excluded from the analysis.

### PI uptake

To determine overall cell death, cells with a disrupted and permeable membrane were detected by addition of propidium iodide (PI). Cells were collected after 72 h, washed with 1× PBS and incubated in 40 µg/ml PI for 10 min. PI positive and thereby dead cells were measured by flow cytometry.

### Measurement of apoptotic cell death by flow cytometry

To define apoptotic cells, measurement of hypodiploid cells was performed as previously described^24^. Alternatively, cell death was determined by staining cells with Annexin-V-FITC and counterstaining with PI^66^. Early apoptotic cells were identified as Annexin-V-FITC positive/PI negative whereas late apoptotic cells display a permeabilized plasma memebrane and consequently are double positive for Annexin-V binding and PI uptake. Analyses were performed using a FACScan (Becton Dickinson, Heidelberg, Germany) and CELLQuest analysis software. Results are given in % of cells.

### Bax/Bak activation

The activation of Bax and Bak was assessed by detecting proteins that undergo conformational change as described before^24^.

### Immunoblotting

Protein expression and phosphorylation status was detected by Western blot analysis. The antibodies α-S6K T389, α-4EBP1 T37/46, α-Bax, α-caspase-8, α-Bcl-2, α-Bcl-x_L_, α-CDK1 T161, α-CDK1 Y15, α-MEK1/2 S217/221 were purchased from Cell Signaling Technology, Inc. (Danvers, MA, USA). The antibodies α-GAPDH, α-Mcl-1, α-Bak were obtained from Santa Cruz Biotechnology, Inc. (Heidelberg, Germany). α-caspase-9 and α-caspase-3 were purchased from R&D Systems, Inc. (Minneapolis, MN, USA). The antibodies α-p53, α-Cyclin B1, and α-Cyclin A were obtained from BD Pharmingen (Heidelberg, Germany). The antibodies α-pHH3 and α-γH2AX were purchased from Sigma-Aldrich (Taufkirchen, Germany). Treated cells were lysed in RIPA buffer^67^, proteins were isolated by centrifugation and protein concentration was measured using the BCA method. SDS-PAGE was performed as described previously^68^. Transfer of proteins onto nitrocellulose membrane was performed with Towbin buffer and wet blot using the Trans-Blot® System from Bio-Rad Laboratories (Hercules, CA, USA). For immunodetection, membranes were incubated with primary antibody at 4 °C overnight. Radish peroxidase labeled secondary antibody was added for 2 h at room temperature and chemiluminescence signal was detected by the use of the ChemiDoc™ MP System (Bio-Rad Laboratories, Hercules, CA, USA).

### JC-1 assay

Loss  of  mitochondrial membrane potential was measured by JC-1 assay as described before^24^.

### siRNA and transfection

Transfection of HCT116 WT and HCT116 Bax/Bak DKO cells was performed using the Amaxa system (Lonza Group AG, Basel, Switzerland). According to Lonza protocol, HCT116 cells were transfected using program D-032 and solution V. To downregulate p53, siRNA, kindly provided from Scheidereit AG (MDC, Berlin, Germany), was used.

### Quantitative RT-PCR

Quantitative PCR was performed as previously described^24^.

### Statistical analysis

Experiments have been performed at least as two independent experiments and results were presented as means ± standard deviation (SD). Where applicable, statistical significance of results was analyzed using Student’s *t*-test.

## Electronic supplementary material

Figure S1

Figure S2

Figure S3

Figure S4

Figure S5

Supplemental Figure Legends
