# Supplementary figures and images for: Pan-class I PI3-kinase inhibitor BKM120 induces MEK1/2-dependent mitotic catastrophe in non-Hodgkin lymphoma leading to apoptosis or polyploidy determined by Bax/Bak and p53
Source: Cell Death Dis. 2018 Mar 7;9(3):384. doi: 10.1038/s41419-018-0413-4 (PMC5841308; doi:10.1038/s41419-018-0413-4)

**A**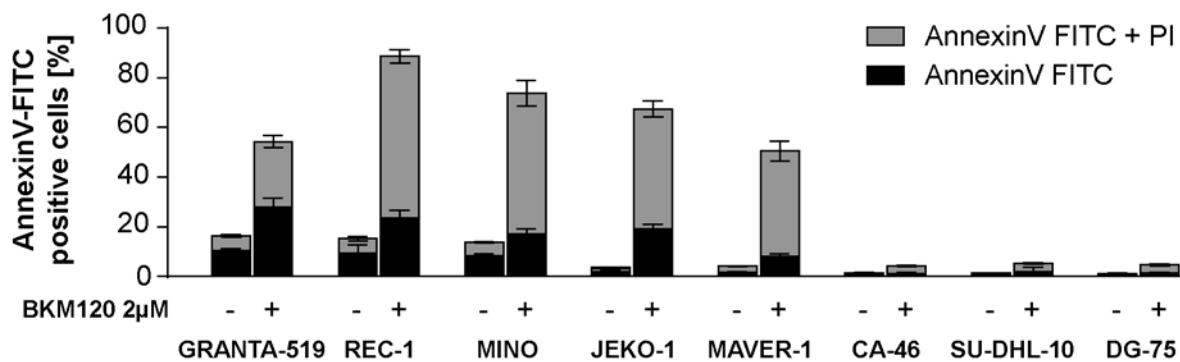**B**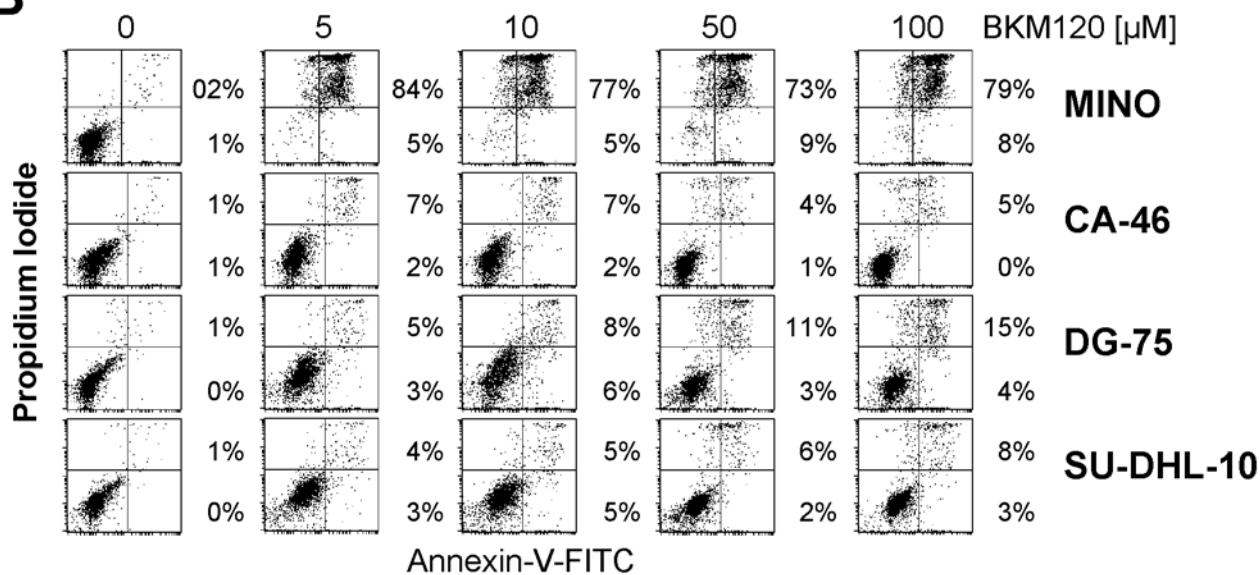**C**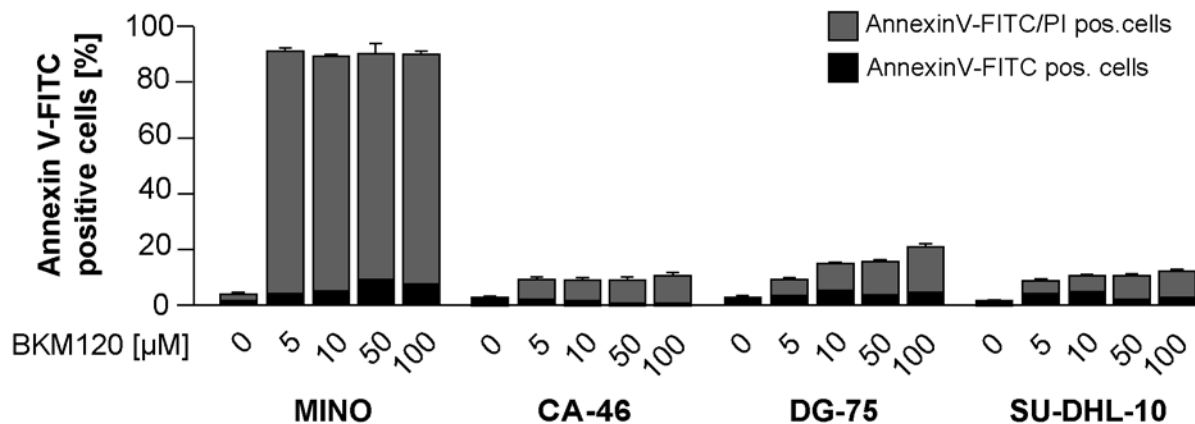**Figure S1**

Supplement: Supplementary file 1 — Figure S1 [file 41419_2018_413_MOESM1_ESM.pdf]

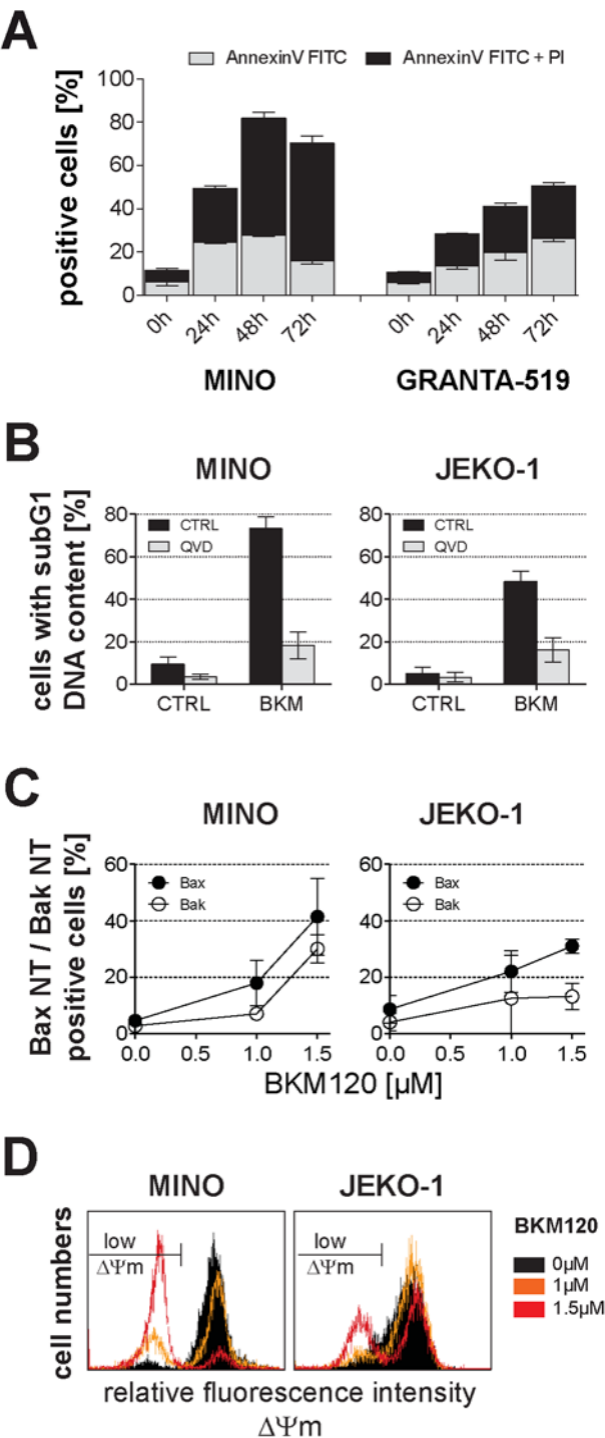

Figure S2

Supplement: Supplementary file 2 — Figure S2 [file 41419_2018_413_MOESM2_ESM.pdf]

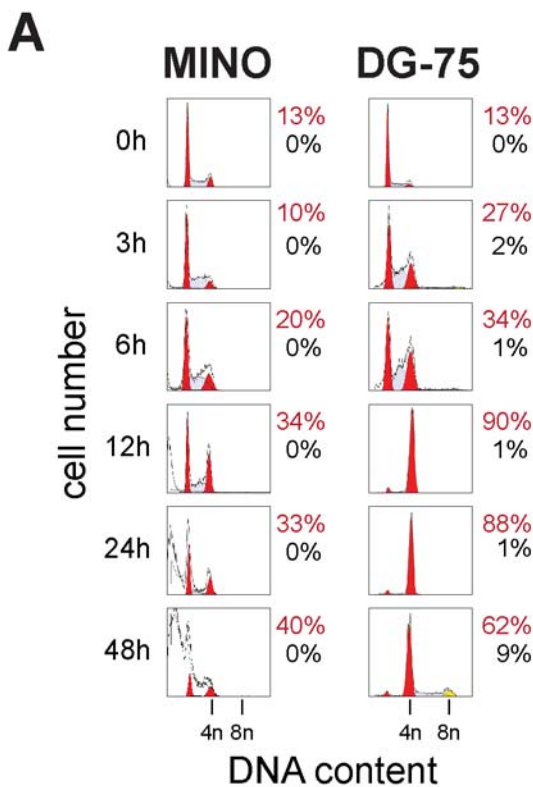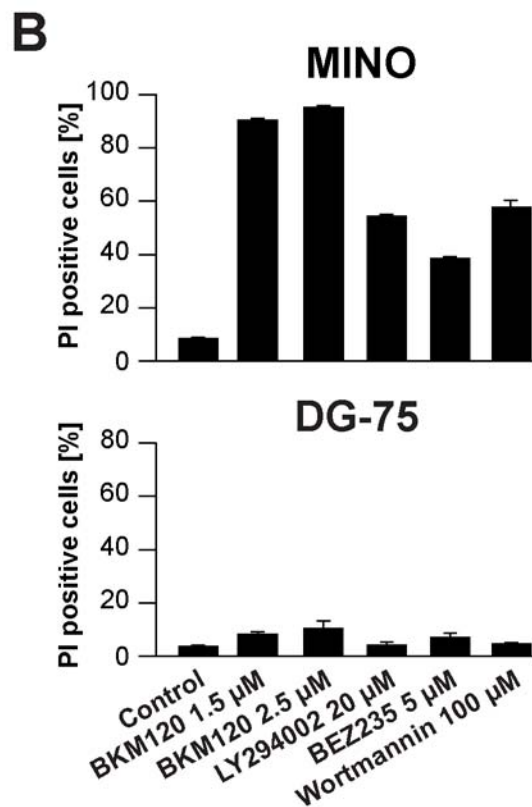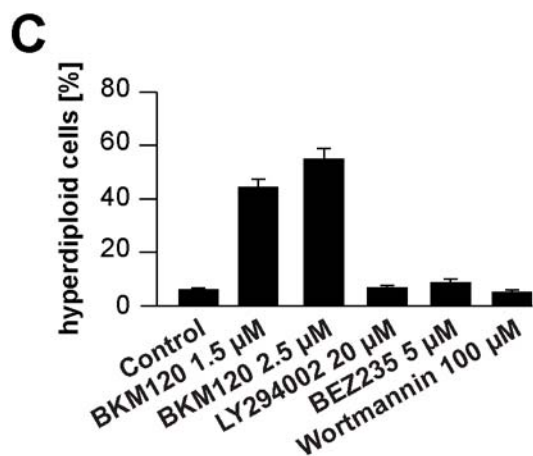

**Figure S3**

Supplement: Supplementary file 3 — Figure S3 [file 41419_2018_413_MOESM3_ESM.pdf]

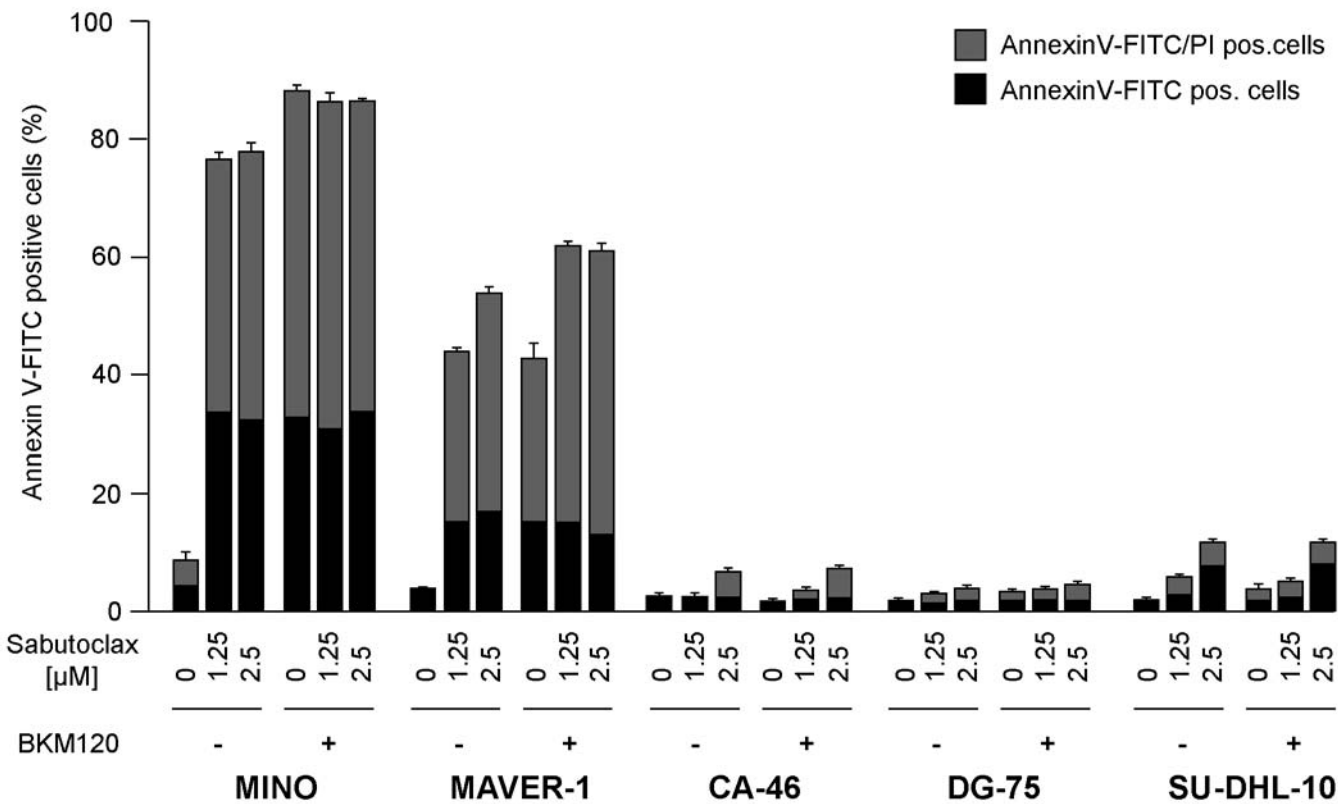

Figure S4

Supplement: Supplementary file 4 — Figure S4 [file 41419_2018_413_MOESM4_ESM.pdf]

**A**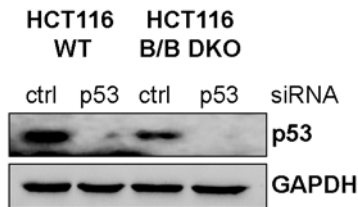**B**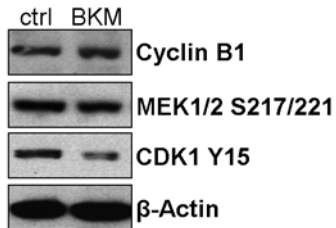**C**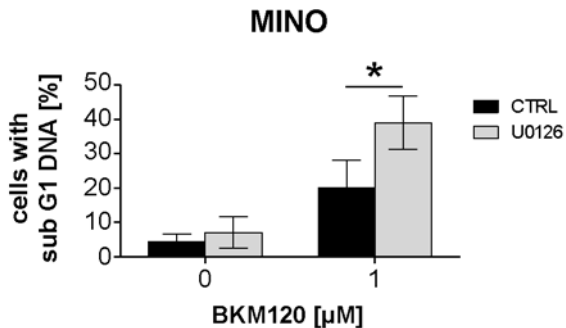**Figure S5**

Supplement: Supplementary file 5 — Figure S5 [file 41419_2018_413_MOESM5_ESM.pdf]
