# Supplementary material for: Pan-class I PI3-kinase inhibitor BKM120 induces MEK1/2-dependent mitotic catastrophe in non-Hodgkin lymphoma leading to apoptosis or polyploidy determined by Bax/Bak and p53
Source: Cell Death Dis. 2018 Mar 7;9(3):384. doi: 10.1038/s41419-018-0413-4 (PMC5841308; doi:10.1038/s41419-018-0413-4)
Supplement: Supplementary file 6 — Supplemental Figure Legends [file 41419_2018_413_MOESM6_ESM.docx]

**Supplemental Figure Legends**

**Supplemental Figure 1**

BKM120 at concentrations up to 100 µM for 72 h did not induce apoptotic cell death in Ca-46, DG-75, and SU-DHL10 cells.

**(A)** Annexin-V-FITC/PI assay of the B-NHL cell line panel after incubation with 2 µM BKM120 for 72 h (bar graph for means +/- SD). **(B)** Annexin-V-FITC/PI assay of MINO, CA-46, SU-DHL-10, and DG-75 cells treated with up to 100 µM BKM120 for 72 h. Representative experiment are shown. Percentages refer to cells positive for both, PI and Annexin-V-FITC staining (upper right quadrant) and cells positive only for Annexin-V-FITC staining (lower right quadrant), respectively. **(C)** Means ± SD from three independent experiments are shown.

**Supplemental Figure 2**

**(A)** Annexin-V-FITC/PI assay of MINO and GRANTA-519 cells after incubation with 2 µM BKM120 for increasing time points. Data shown are representative for three independent experiments. **(B)** Measurement of Sub-G1 cells in JEKO-1 and MINO in response to 1.5 µM BKM120 for 72 h and concomitant blockage of caspases with 10 µM QV-D-OPh. Means ± SD from three independent experiments are shown**. (C)** Conformational changes of Bax and Bak after treatment of JEKO-1 and MINO with increasing concentration of BKM120 for 48 h. Activation of Bax and Bak is accompanied by a conformational change leading to the exposure of the respective N-terminus that is inaccessible in vital cells. To study Bax or Bak activation a flow cytometric immunofluorescence analysis by use of conformation-specific antibodies directed against the respective N-terminus was performed. Means ± SD from three independent experiments are shown. **(D)** Loss of mitochondrial membrane potential in JEKO-1 and MINO after incubation with 0 µM (black), 1 µM (orange) or 1.5 µM (red) BKM120 for 48 h. Data shown are representative for three independent experiments.

**Supplemental Figure 3**

**(A)** Cell cycle distribution of MINO and DG-75 incubated with 1.5 µM BKM120 for the indicated time points. Quantification with ModFit LT. Data are representative for three independent experiments. (B) MINO (upper panel) and DG-75 (lower panle) cells were treated with different PI3 kinase inhibitors for 72 h. Cell death induction was assessed using PI uptake. (C) Analysis of cellular DNA content in DG-75 cells revealed that BKM120 but none of the other inhibitors induces polyploidy in DG-75 cells. (mean ± standard deviation (SD) of three independent experiments)

**Supplemental Figure 4**

Sabutoclax did not induce apoptosis in BKM120 resistant cell lines or sensitize these cells for BKM120-induced cell death (mean ± standard deviation (SD) of for three independent experiments).

**Supplemental Figure 5**

**(A)** Protein expression of p53 after ctrl siRNA or p53 siRNA transfection of HCT116 WT or HCT 116 Bax^-/-^/Bak^-/-^ cells. **(B)** Western blot analysis of HCT116 cells showed constitutive, BKM120 independent, MEK1/2 activation and reduced CDK1 Y15 phosphorylation upon BKM120 treatment. **(C)** Apoptosis of MINO cells, incubated with 1 µM BKM120 alone or in combination with 50 µM U0126, was determined by measuring percentages of cells with a hypodiploid DNA content (sub G1 cells). Mean and SD of for three independent experiments. * p<0.05.
